# How to Assess the Functional Impact of Atrial Fibrillation in Patients With Heart Failure

**DOI:** 10.1002/clc.70140

**Published:** 2025-04-30

**Authors:** Naoya Kataoka, Teruhiko Imamura

**Affiliations:** ^1^ Second Department of Internal Medicine University of Toyama Toyama Toyama Japan

**Keywords:** ablation, electrocardiogram, quality of life

To the Editor,

A well‐established interplay exists between atrial fibrillation (AF) and heart failure (HF). However, the specific adverse impact of AF on quality of life among HF patients remains incompletely elucidated. The authors demonstrated that the presence of AF and reduced ejection fraction was associated with a lesser degree of improvement in functional class and the physical dimension of a quality‐of‐life questionnaire [1]. Several concerns merit consideration.

In the present study, patients with HF were included regardless of their left ventricular ejection fraction (LVEF) [1]. Prior studies focusing on HF populations over 60 years of age have shown that quality‐of‐life profiles and depressive symptoms differ significantly between cohorts with preserved versus reduced LVEF [2]. To better delineate the target population, it may be more appropriate to restrict the LVEF range.

The study did not differentiate between paroxysmal and persistent forms of AF [1]. Patients with paroxysmal AF may experience a more favorable quality of life, particularly when sinus rhythm is intermittently or spontaneously restored [3]. Stratification by AF subtype and burden may provide deeper insight into the differential impact on patient‐reported outcomes.

The definition of arrhythmia‐induced cardiomyopathy warrants clarification. This condition is typically diagnosed by the observation of reverse remodeling following the resolution of the causative arrhythmia [4]. It may be reasonable to consider arrhythmia‐induced cardiomyopathy within the broader category of idiopathic cardiomyopathies, given its distinct pathophysiological trajectory.

In the current era, catheter ablation has emerged as a standard therapeutic option for AF in patients with HF [5]. Notably, in the present study, great improvement in New York Heart Association functional class was achieved with pharmacological therapy alone. A discussion regarding the therapeutic approach—namely, rhythm control versus rate control—would enhance the clinical relevance of the study's findings in the context of contemporary HF management.

## Ethics Statement

The authors have nothing to report.

## Consent

The authors have nothing to report.

## Conflicts of Interest

The authors declare no conflicts of interest.

## Data Availability

The authors have nothing to report.
